# CK1α agonists attenuate medulloblastoma stemness and relapse risk

**DOI:** 10.1038/s41419-026-08762-6

**Published:** 2026-04-24

**Authors:** Kendell Peterson, Maria Turos-Cabal, Pritika Shahani, April D. Salvador, Marzena Swiderska-Syn, Giulia D. S. Ferretti, Carlos Alfaro-Quinde, Valentin Kliebe, Laura Finelli, Ashley J. Howell, Megan E. Vieira, Isabel Palomo-Caturla, Dennis L. Fei, Daniel T. Wynn, Vanesa Martin, Thibaut Barnoud, Jezabel Rodriguez-Blanco

**Affiliations:** 1 Darby Children’s Research Institute, Department of Pediatrics, Medical University of South Carolina, Charleston, SC USA; 2 Department of Biochemistry and Molecular Biology, Medical University of South Carolina, Charleston, SC USA; 3 StemSynergy Therapeutics Inc., Miami, FL USA; 4 Department of Oncology, Lombardi Comprehensive Cancer Center, Georgetown University, Washington DC, USA; 5 University Institute of Oncology of the Principality of Asturias, University of Oviedo, Oviedo, Spain; 6 Hollings Cancer Center, Medical University of South Carolina, Charleston, SC USA

**Keywords:** CNS cancer, Cancer stem cells

## Abstract

While outcomes for most children with medulloblastoma (MB) are relatively favorable, those in the Sonic Hedgehog (SHH) subgroup with *Tumor protein P53* (*TP53*) mutations—known as the SHHα subtype—face a much poorer prognosis. SHHα patients relapse more frequently and rapidly, underscoring the need for therapies that prevent recurrence. We recently identified a non-canonical Gli-driven Sox2⁺ cell population that promotes relapse in SHH MB. However, few Gli-targeting strategies have shown clinical promise to date. One translational Gli inhibitor is pyrvinium, an FDA-approved compound known to destabilize Gli through increasing Casein kinase 1α (CK1α) activity. In this study, we tested whether pyrvinium and a brain-permeable derivative, SSTC3, affect stemness and relapse risk in mouse and human-derived SHHα MB models. We found that pyrvinium suppresses the Gli-driven proliferation of Sox2⁺ cells. Unlike other SHH/Gli-targeting approaches, pyrvinium also impaired MB self-renewal by depleting Cluster of Differentiation 15 (CD15)⁺ cells. Mechanistic studies revealed that CD15⁺ cell self-renewal is WNT-dependent and driven by the loss of p53/*microRNA-34a*-mediated repression of WNT signaling. Remarkably, pyrvinium and SSTC3 reduced Sox2⁺, CD15⁺, and dual Sox2/CD15-labeled populations in mouse and patient-derived SHHα models. Consistent with their ability to diminish tumor stemness, pyrvinium also impaired primary and secondary tumor engraftment. Our findings show that CK1α agonists regulate stemness in SHHα MB, establishing CK1α as a therapeutically relevant vulnerability. While pyrvinium itself is not an ideal clinical candidate, these data support the development of second-generation brain-penetrant CK1α-targeting derivatives.

## Introduction

Brain tumors are the leading cause of cancer-related death in children, with medulloblastoma (MB) being the most common malignant form [1]. From the four major molecular subgroups, the Sonic Hedgehog (SHH) subgroup accounts for ~30% of cases [2]. Clinical outcomes in this subgroup are strongly influenced by co-occurring genetic alterations. In particular, *Tumor protein P53* (*TP53*) mutations, present in ~7–30% of cases at diagnosis [3–5] and ~30% at recurrence [4], are associated with increased relapse risk and shorter time to relapse [3], and thus these tumors are classified as very high-risk [6]. Given their dismal outcomes, there is an urgent need to understand the mechanisms driving relapse in *TP53*-mutant SHH MB, also known as SHHα [7].

Emerging evidence suggests that a rare population of MB progenitor cells (MPCs), often marked by the stemness factor SRY-box transcription factor 2 (Sox2), may contribute to disease recurrence [8]. Our previous work in SHH MB showed that the expansion of Sox2⁺ cells relies on non-canonical Gli activity, rendering them resistant to Smoothened (Smo) inhibitors in clinical development [9]. By contrast, compounds that block Gli-dependent transcription via Bromodomain and extra-terminal (BET) inhibition reduced Sox2⁺ cells and relapse risk in SHH MB animal models [9]. Nevertheless, the toxicity associated with BET inhibitors in clinical settings [10] underscores the need for alternative Gli-targeting approaches to prevent MB recurrence.

One translational approach to target Gli involves activating Casein kinase 1α (CK1α). CK1α is a serine/threonine kinase that phosphorylates Gli, promoting its degradation [11]. Small-molecule CK1α agonists, such as the FDA-approved anti-helminthic drug pyrvinium, selectively bind CK1α and function as allosteric activators [12]. Through CK1α activation, pyrvinium and a brain-permeable derivative, SSTC3, attenuate SHH-driven MB growth [13, 14]. However, CK1α also phosphorylates β-catenin, an essential transcriptional activator in the Wingless-related integration site (WNT) pathway, targeting it for proteasomal degradation [15]. Thus, CK1α agonists extend their effect beyond Gli, as both pyrvinium and SSTC3 block WNT signaling and suppress the growth of WNT-driven malignancies [16, 17].

Given their dependence on Gli signaling, we hypothesized that CK1α agonists deplete Sox2⁺ cells implicated in SHHα MB relapse. Here, we show that CK1α agonists target not only Gli-driven Sox2^+^ cells but also a WNT-driven, self-renewing CD15^+^ compartment, thereby limiting the ability of residual disease to re-engraft. Together, these findings support CK1α modulation as a strategy to suppress relapse-driving cell populations in very high-risk SHHα MB.

## Materials and methods

### Mouse studies

All animal work was conducted in compliance with the NIH Guide for the Care and Use of Laboratory Animals. Experimental protocols received approval from the Institutional Animal Care and Use Committees of the Medical University of South Carolina and the University of Miami. *Ptch1*^*tm1Mps*^*/J* (*Ptch1-LacZ*) and *B6.129S2-Trp53*^*tm1Tyj*^*/J* (*Trp53-KO*) mice (Jackson Laboratory) were crossed to establish a colony. Spontaneous tumors from either female or male mice were expanded and maintained as allografts in 6–10-week-old *CD1-Foxn1*^*nu*^ male mice (Charles River). For pyrvinium (Enzo or Sigma-Aldrich) treatment, 1 × 10^6^ viable cells (trypan blue-excluded) were implanted subcutaneously (s.c.) into similar mice. Once tumors reached ~200 mm³, mice received pyrvinium (0.8 mg/kg, s.c.) near the tumor every other day (q.o.d.). Tumors were harvested 6 hours (h) after the final dose and either fixed or dissociated with Papain (Worthington) or Accutase (Invitrogen) for fluorescence-activated cell sorting (FACS), sphere formation, or re-engraftment assays.

For flank re-engraftment, indicated viable cells were implanted s.c. into 6–10 weeks old *CD1-Foxn1*^*nu*^ female or male mice. For orthotopic re-engraftment, 1 × 10^4^ viable cells from pyrvinium-treated tumors were resuspended in 3 µL Neurobasal-A media and implanted into the cerebellum [18]. For primary engraftment, MPC-2 cultures were treated with 200 nM pyrvinium for 24 h before s.c. implantation.

For SSTC3 (StemSynergy Therapeutics) studies, 6–10-week-old *CD1-Foxn1*^*nu*^ and NOD.Cg-Prkdc^scid^ Il2rg^tm1Wjl^/SzJ male mice were orthotopically implanted with 1 × 10^5^ *Ptch1-LacZ; Trp53-KO* cells or 1 × 10^6^ *TP53*-mutant patient-derived xenograft (PDOX) cells (SJSHHMB-14-7196, courtesy of Dr. Roussel, St. Jude), respectively. Ten- and thirty-day post-implantation, respectively, mice received daily (q.d.) intraperitoneal (i.p.) injections of vehicle or SSTC3 (10 mg/kg) for 3 days. Brains were then harvested for flow cytometry or IHC.

### Statistical analysis

Results represent the mean ± SEM from at least three independent experiments. For BrdU staining, four fields per condition from three experiments were quantified. Multiple group comparisons used one-way ANOVA with post hoc Dunnett analysis. Two-sample comparisons used one-tailed Student’s *t* tests. Symptom-free survival was assessed using Log-rank (Mantel–Cox) tests. Tumor engraftment significance was determined using a *χ*² test. Statistical tests were selected as appropriate for each dataset and are indicated in the corresponding figure legends. Data distribution and variance were evaluated before analysis to confirm suitability for parametric testing, and variance was comparable between groups. For in vivo studies, the exact number of biological replicates is indicated in each panel, either by individual dots representing independent tumors or by listing the number. Mice were randomized before dosing and sample size was based on our prior experience with similar models [9, 14, 18]. There was no blinding during in vivo experiments. Statistical significance: *P < 0.05, **P < 0.01, ***P < 0.001, ****P < 0.0001.

Additional Materials and methods are described in Supplemental Methods.

### Ethics approval and consent to participate

No human subjects were involved in this study. All animal experiments were conducted in compliance with the National Institutes of Health (NIH) Guide for the Care and Use of Laboratory Animals. Experimental protocols were reviewed and approved by the Institutional Animal Care and Use Committees (IACUC) at the Medical University of South Carolina (IACUC-2019-00870-1) and the University of Miami (13-217).

## Results

### Pyrvinium blocks Gli1-driven Sox2^+^ cell proliferation

**Fig. 1:**
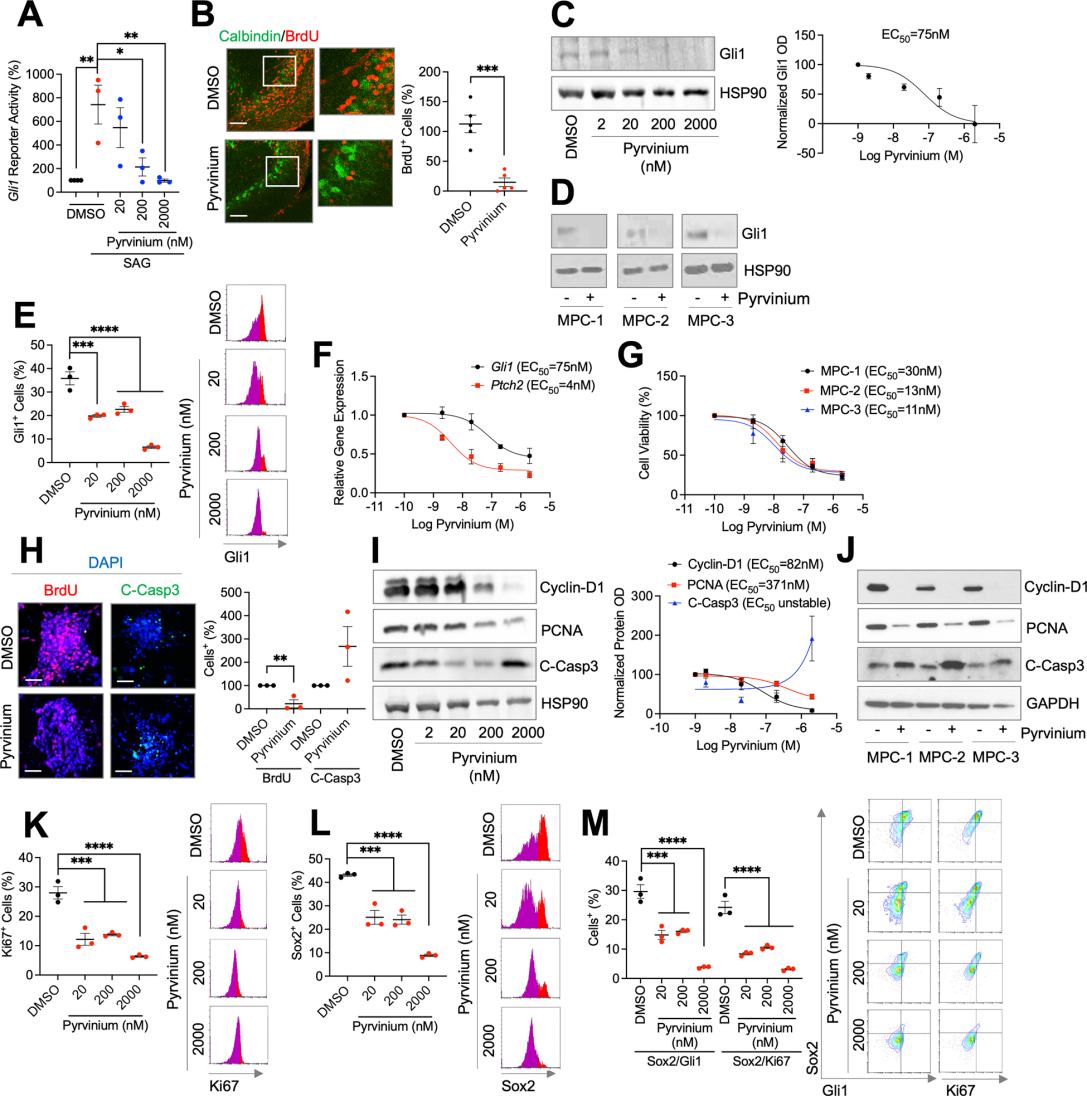
Pyrvinium attenuates SHH signaling in MPC cultures. **A** SHH signaling was induced in *Gli1* reporter cells with 100 nM SAG for 24 h, followed by treatment with the indicated concentrations of pyrvinium for an additional 24 h. **B** Organotypic cerebellar slices from 7-10-day-old mice were treated with 200 nM pyrvinium for 24 h, followed by a BrdU pulse. Quantification of BrdU^+^ cells is shown. Data were analyzed using an unpaired *t* test. Calbindin was used to label the Purkinje layer. **C** Gli1 levels were quantified by immunoblotting in MPC-2 cultures treated with increasing concentrations of pyrvinium for 24 h (OD: optical density). **D** Gli1 levels were similarly evaluated in the indicated MPC cultures treated with 200 nM pyrvinium for 24 h. **E** MPC-1 cultures were treated with the indicated concentrations of pyrvinium for 24 h, and Gli1-labeled cells were quantified by flow cytometry. **F** Expression of SHH target genes was measured by RT-qPCR in MPC-2 cultures treated with increasing concentrations of pyrvinium for 24 h. **G** MPC cultures were treated with pyrvinium for 72 h, and cell viability was assessed using an MTT assay. **H** MPC-2 cultures were exposed to 200 nM pyrvinium for 24 h before assessing BrdU incorporation and cleaved Caspase-3⁺ (C-Casp3). Quantification of positive cells is shown. Data was analyzed using an unpaired *t* test. **I** Levels of the indicated proteins were quantified in extracts from MPC-2 cultures treated with increasing concentrations of pyrvinium for 24 h. **J** Levels of the indicated proteins were assessed in extracts from the indicated MPC cultures treated with 200 nM pyrvinium for 24 h. **K** MPC-1 cultures were treated with the indicated concentrations of pyrvinium for 24 h, and Ki67-labeled cells were quantified by flow cytometry. **L** MPC-1 cultures were exposed to the indicated concentrations of pyrvinium, and numbers of Sox2⁺ cells were determined by flow cytometry. **M** MPC-1 cultures were exposed to the indicated concentrations of pyrvinium, and numbers of Sox2⁺/Gli1⁺ and Sox2⁺/Ki67⁺ cells were determined by flow cytometry. In all cases, representative IF images (scale bar 100 µm) and flow cytometry plots are shown. Unless otherwise indicated, mean ± SEM of data normalized to DMSO were analyzed using a one-way ANOVA followed by Dunnett’s post hoc. All EC_50_s were calculated using non-linear regression analyses. *P < 0.05, **P < 0.01, ***P < 0.001, ****P < 0.0001.

We previously showed that the propagation of MB cultures enriched in Sox2^+^ cells, herein referred to as MPC cultures, is driven by non-canonical Gli activation downstream of Smo [9]. Because CK1α destabilizes Gli [11], we tested the efficacy of pyrvinium, a known CK1α agonist [12], in MPC cultures. Given that most SHH MB relapses are associated with *TP53* mutations [3, 5], we focused on *Trp53*-deficient cultures (*Trp53-KO*). According to its mechanism of action, pyrvinium not only reduced *Gli1*-driven promoter activity in a reporter cell line (Fig. 1A) but also decreased the SHH-driven cell proliferation of the granule layer of the developing cerebellum in organotypic cultures (Fig. 1B and Supplemental Fig. 1A). In MPC cultures, nanomolar concentrations of pyrvinium reduced Gli1 levels (Fig. 1C, D) and the number of Gli1⁺ cells (Fig. 1E), thereby attenuating the expression of SHH target genes (Fig. 1F). Furthermore, pyrvinium reduced the number of viable cells in MPC cultures, with an average EC50 of 17 nM (Fig. 1G), which is consistent with their dependency on Gli to propagate [9].

Given pyrvinium’s ability to reduce the number of viable cells in MPC cultures, we investigated whether this effect was due to decreased proliferation or increased cell death. BrdU incorporation assays suggested that pyrvinium’s effects are primarily attributed to reduced proliferation, as evidenced by a drop in BrdU^+^ cells (Fig. 1H and Supplemental Fig. 1B). This was further supported by the reduction in levels of proliferation markers, such as Cyclin-D1 and PCNA (Fig. 1I, J), as well as cells labeled for the proliferation marker Ki67 (Fig. 1K). In contrast, pyrvinium had only minimal effects on cell death, as determined by the number of cleaved Caspase-3-labeled cells (Fig. 1H and Supplemental Fig. 1B) and its protein levels (Fig. 1I, J). Importantly, similar to other Gli inhibitors [9], pyrvinium also reduced the number of Sox2⁺ cells in MPC cultures (Fig. 1L), including those Sox2^+^ cells co-labeled with Gli1 and Ki67 (Fig. 1M). Together, these data suggest that pyrvinium depletes Sox2⁺ cells by blocking their Gli-driven proliferation. Supporting a non-canonical Gli activation downstream of Smo, a compound acting on Smo, vismodegib, had little effect on the levels of Gli1 in Sox2^+^ cell-enriched cultures (Supplemental Fig. 1C). Subsequently, vismodegib neither affected the levels of proliferation and cell death markers (Supplemental Fig. 1D), or the specific numbers of Sox2^+^ cells (Supplemental Fig. 1E) in these cultures.

### Pyrvinium attenuates MB self-renewal

**Fig. 2:**
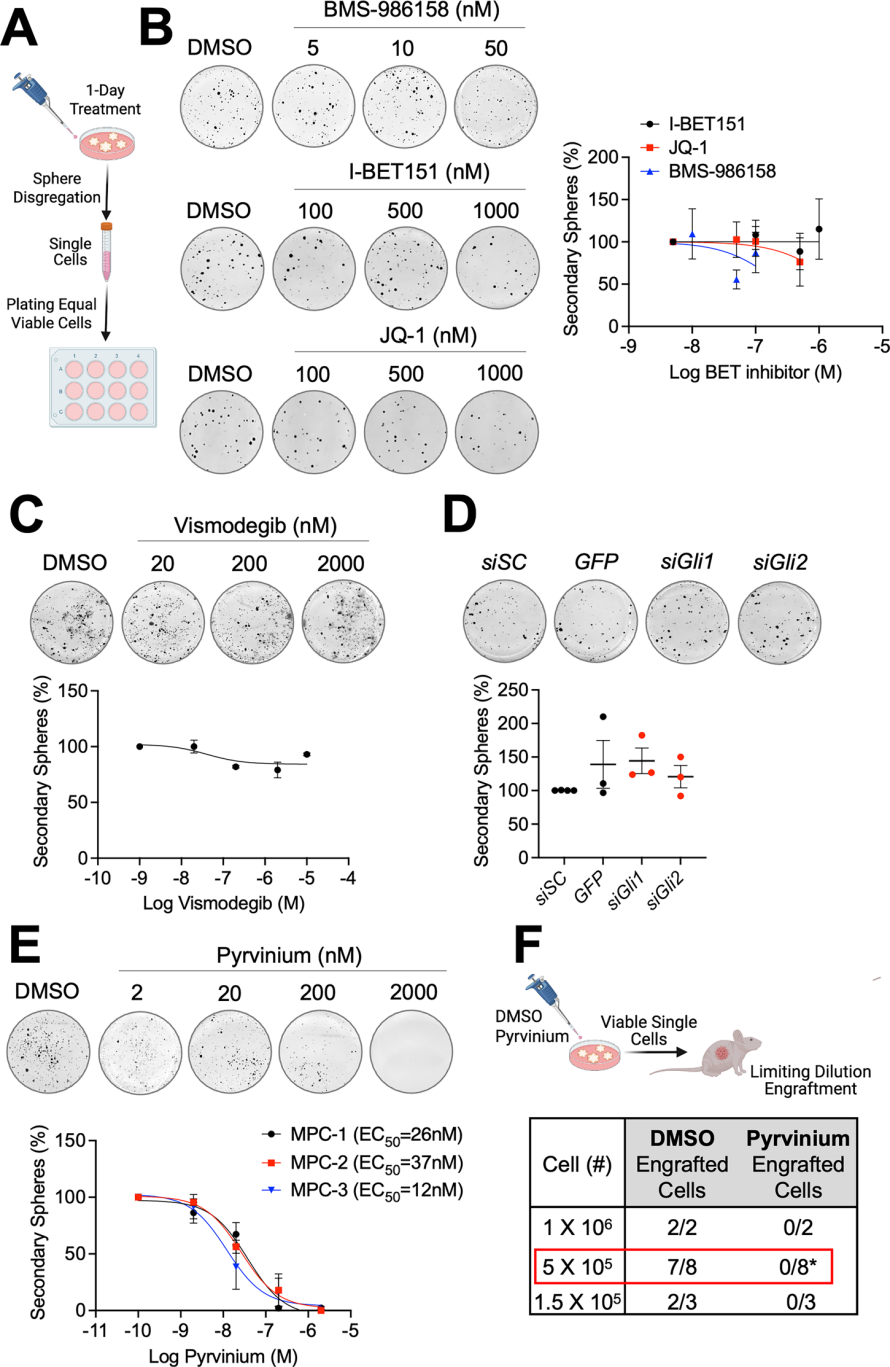
Pyrvinium attenuates MB self-renewal. **A** Schematic of the self-renewal protocol. **B** MPC-2 cultures were exposed to increasing concentrations of indicated BET inhibitors for 24 h before allowing equal numbers of viable cells to form new spheres for one week. **C** Similar cultures were exposed to increasing concentrations of vismodegib before completing self-renewal protocols. **D** MPC-2 cultures were transfected with siRNA pools targeting *Gli1* and *Gli2*, as well as scramble *siRNA* (*siSC*) and GFP-labeled controls. Seventy-two hours after transfection, spheres were dissociated, and equal numbers of viable cells were replated. A one-way ANOVA was used to analyze data normalized to *siSC*. **E** MPC cultures were allowed to form secondary spheres following a 24 h incubation with the indicated concentrations of pyrvinium. **F** Indicated numbers of viable cells from MPC-2 cultures treated with 200 nM pyrvinium for 24 h were subcutaneously implanted. The frequency of tumor engraftment was analyzed using a one-sided *χ*^2^ test. In all cases, representative images of self-renewal assays are shown. Unless otherwise indicated, mean ± SEM of data normalized to DMSO were analyzed using non-linear regression tests. *P < 0.05, **P < 0.01, ***P < 0.001, ****P < 0.0001.

MPCs are known to self-renew to maintain tumor stemness [19]. Interestingly, while our previous work showed that Gli inhibition by targeting BET proteins reduced MPC culture proliferation [9], the ability of these drugs to block secondary sphere formation, used as a measure of tumor stemness [20], was not tested at that time. To evaluate this, we used self-renewal protocols in which MPC cultures were exposed to drugs of interest for 24 h before disaggregating spheres [18]. Equal numbers of viable cells were next re-plated and allowed to form new spheres in the absence of drug (Fig. 2A). These studies revealed that three structurally distinct BET inhibitors (I-BET151, JQ-1, BMS-986158) fail to block the self-renewal of MPC cultures (Fig. 2B) at concentrations we previously showed to attenuate SHH signaling and reduce MPC viability [9]. Similarly, the Smo inhibitor vismodegib failed to impair MPC secondary sphere formation (Fig. 2C), suggesting that MPC self-renewal is not SHH/Gli-driven. Accordingly, siRNA pools targeting *Gli1* and *Gli2* also failed to inhibit MB self-renewal (Fig. 2D). In contrast, pyrvinium attenuated secondary sphere formation in MPC cultures, with an average EC50 of 25 nM (Fig. 2E). Given pyrvinium’s effect on MB self-renewal ex vivo, we studied whether it similarly affects tumor initiation. MPC cultures were exposed to pyrvinium before implanting limiting dilutions of viable cells into mice. Tumor initiation was observed from the vehicle-treated cultures, even when only 150,000 cells were implanted. However, pyrvinium-treated cells did not form tumors, even when nearly tenfold higher cell numbers were used (Fig. 2F). Together, these findings suggest that pyrvinium blocks MB self-renewal by targeting a pathway different than SHH/Gli.

### Pyrvinium inhibits MB self-renewal by targeting WNT signaling

**Fig. 3:**
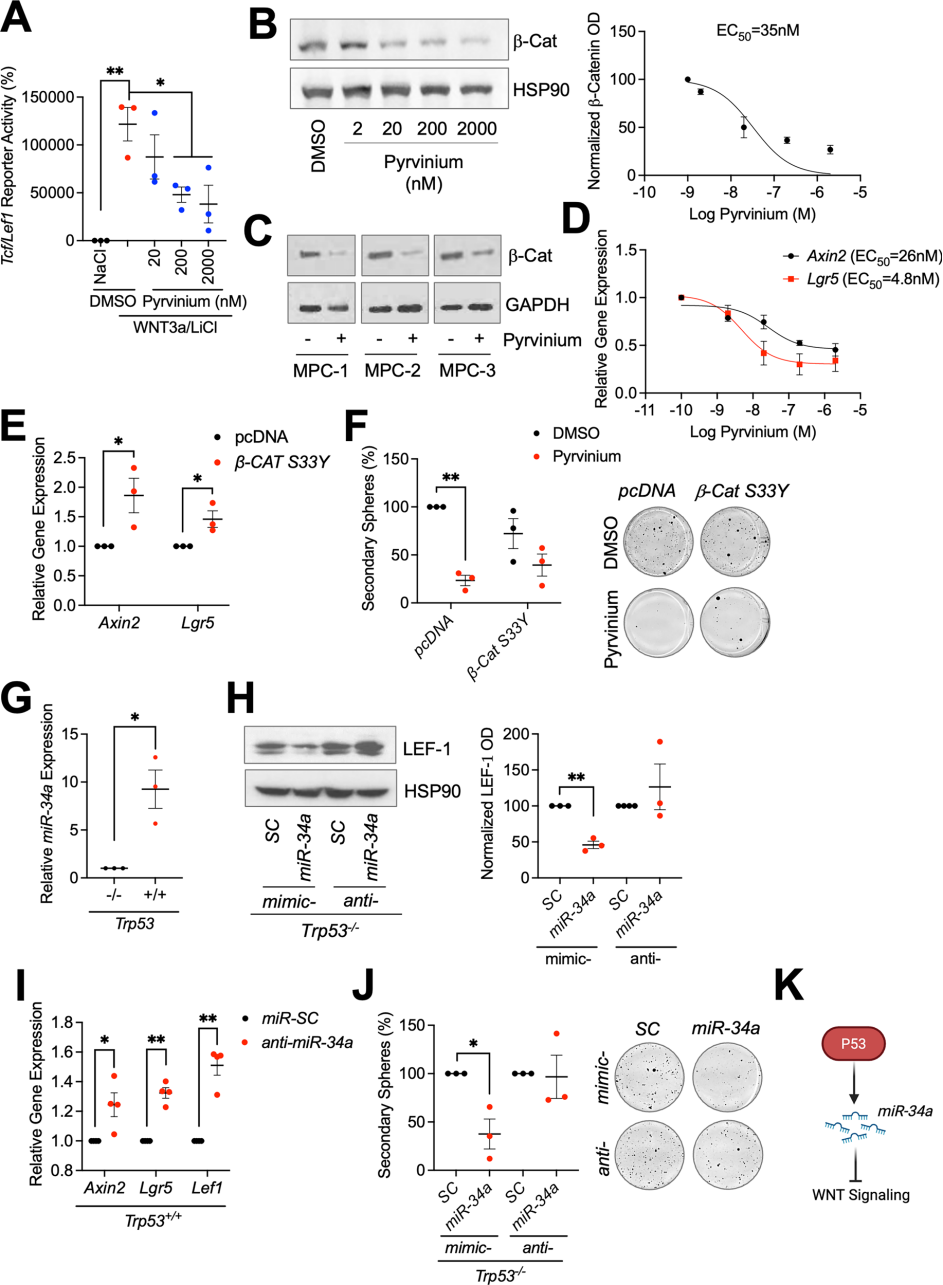
Pyrvinium acts on WNT signaling to block MB self-renewal. **A** WNT signaling was induced in TCF/LEF1 reporter cells by 100 ng/ml WNT3a and 10 mM LiCl for 24 h before treatment with pyrvinium for an additional 24 h. Data was analyzed using a one-way ANOVA followed by Dunnett’s post hoc. **B** The levels of β-catenin following 24 h incubation with increasing concentrations of pyrvinium were quantified in MPC-2 cultures by immunoblotting. (OD: optical density). **C** The levels of β-catenin following 24 h incubation with 200 nM pyrvinium were similarly determined in the indicated MPC cultures. **D** MPC-2 cultures were exposed to indicated concentrations of pyrvinium for 24 h before determining the expression of indicated WNT target genes by RT-qPCR. **E** MPC-2 cultures were transfected with the indicated vectors and expression of WNT target genes was determined 48 h later. Data was normalized to *pcDNA* control. **F** MPC-2 cultures were transfected with the indicated vectors. 3 days post-transfection cells were treated with 200 nM pyrvinium for an additional 24 h before sphere dissociation and re-plating of equal numbers of viable cells. Numbers of secondary spheres were quantified 7 days later. Data was normalized to *pcDNA* control. **G** The expression of *miR-34a-5p* was determined in MPC-2 (*Trp53*^−/−^) and MPC-47 (*Trp53*^+/+^) *Ptch1-LacZ* cultures by RT-qPCR and normalized to MPC-2 data. **H** MPC-2 cultures were transfected with specific *mimic-* or *anti-miR* sequences along with respective scramble (*miR-SC*) controls, and LEF1 levels were quantified by immunoblotting 48 h later. **I** MPC-47 cells were transfected with *anti-miR-34a* or a scramble control, and the expression of WNT target genes was determined 48 h later by RT-qPCR. Results were normalized to the corresponding scramble sequence. **J** MPC-2 cultures were transfected with indicated *mimic*- and *anti-miR* sequences, and secondary sphere formation was determined 72 h later. Results were normalized to the corresponding scramble sequence. **K** Schematic of the mechanism of activation of WNT signaling in *Trp53* mutant MPC cultures. In all cases, representative images of self-renewal assays are shown. Unless otherwise indicated, mean ± SEM data were analyzed using an unpaired *t* test. All EC_50_s were calculated using non-linear regression analyses. *P < 0.05, **P < 0.01, ***P < 0.001, ****P < 0.0001.

In addition to regulating Gli stability [11], CK1α is also known to prime β-catenin for degradation [15], thereby suppressing WNT signaling. Moreover, our previous findings indicated that self-renewal in *Trp53-KO* MB is driven by WNT activation [18]. Thus, we wondered whether pyrvinium’s effects on self-renewal operate through WNT inhibition. Supporting this idea, pyrvinium reduced WNT activity in a TCF/LEF1 reporter assay (Fig. 3A) and decreased both β-catenin levels (Fig. 3B, C) and expression of WNT target genes (Fig. 3D) in MPC cultures. Furthermore, overexpression of a constitutively active β-catenin form (*S33Y* mutant) increased baseline expression of WNT biomarkers (Fig. 3E) and reduced pyrvinium’s efficacy on MPC self-renewal (Fig. 3F), supporting a WNT-dependent mechanism of action. Further supporting a WNT-mediated self-renewal, vismodegib, which we showed does not alter MPC self-renewal, likewise failed to attenuate WNT signaling (Supplemental Fig. 1F).

Although our prior work linked WNT activation to p53 loss in MPC cultures [18], the mechanism driving elevated WNT signaling remained unclear. One possible explanation involves *microRNAs* (*miRNAs*), small non-coding RNAs that bind to 3’ UTRs of target *mRNAs* and suppress their translation [21]. p53 loss has previously been associated with downregulation of *miR-34a*, leading to de-repression of WNT components such as LEF1 [22], a key member of the β-catenin transcriptional complex [23]. Accordingly, *Trp53-KO* MPC cultures exhibited reduced levels of *miR-34a* compared to wild-type counterparts (Fig. 3G). Introduction of a *mimic-miR-34a* into *Trp53-KO* MPC cultures decreased LEF1 levels, whereas inhibition of *miR-34a* using an *anti-miR* did not significantly increase LEF1 levels at a similar timepoint (Fig. 3H). However, an *anti-miR-34a* increased WNT target gene expression in *Trp53-wild-type* MPCs (Fig. 3I). Further implicating *miR-34a* in MB self-renewal, a *mimic-miR-34a* reduced secondary sphere formation in *Trp53-KO* MPC cultures, while an *anti-miR-34a* had minimal effect (Fig. 3J). These findings suggest that loss of p53 reduces *miR-34a* levels in MPC cultures, thereby enabling WNT activation (Fig. 3K).

### Pyrvinium depletes a self-renewing CD15^+^ cell population

**Fig. 4:**
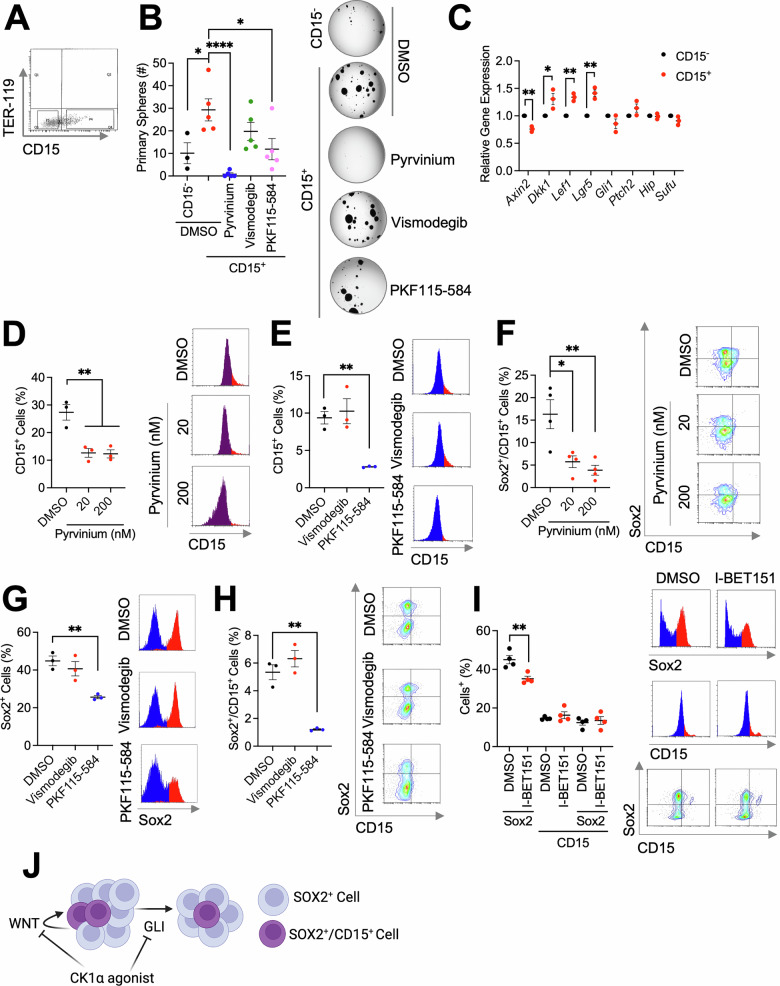
Pyrvinium depletes a self-renewing CD15^+^ cell population. **A** CD15⁺/Ter-119⁻ cells were flow-sorted from *Ptch1-LacZ, Trp53-KO* MB tumors. **B** Similarly sorted cells were plated in the presence of vismodegib (200 nM), PKF115-584 (1000 nM), or pyrvinium (200 nM), and their ability to form spheres was determined. **C** RNA was extracted from CD15⁺/Ter-119⁻ and CD15⁻/Ter-119⁻ sorted pools, and expression of WNT and SHH target genes was determined by RT-qPCR. Data were normalized to CD15⁻ cells and analyzed using an unpaired *t* test. **D** MPC-1 cultures were exposed for 24 h to increasing concentrations of pyrvinium and numbers of CD15⁺ cells were determined by flow analysis. **E** Similar cultures were exposed to vismodegib (200 nM) or PKF115-584 (1000 nM) for 24 h, and CD15⁺ cell numbers were determined by flow analysis. **F** The number of Sox2^+^/CD15^+^ cells was determined by flow cytometry in MPC-1 cultures treated with increasing concentrations of pyrvinium for 24 h. **G** Similar cultures were exposed to vismodegib (200 nM) or PKF115-584 (1000 nM) for 24 h, and Sox2⁺ cell numbers were determined by flow analysis. **H** The number of Sox2^+^/CD15^+^ cells was determined by flow cytometry in similarly treated cultures. **I** MPC-1 cultures were exposed to I-BET151 (500 nM) for 24 h, and the numbers of indicated positive cells were determined by flow analysis. Data was analyzed using an unpaired *t* test. **J** A schematic of the mechanism of action of pyrvinium in MPC cultures. Unless otherwise indicated, mean ± SEM of data normalized to DMSO were analyzed using a one-way ANOVA followed by Dunnett’s post hoc. *P < 0.05, **P < 0.01, ***P < 0.001, ****P < 0.0001.

The ability of BET inhibitors to deplete Sox2 cells [9] without affecting self-renewal suggests the involvement of an alternative cell pool. To define the self-renewing compartment targeted by pyrvinium, we focused on CD15⁺ cells, which were previously shown to represent a tumor-initiating population in SHH MB [24–26]. CD15⁺ cells were sorted from *Ptch1-LacZ, Trp53-KO* tumors, using Ter-119 to deplete erythroid lineage cells (Fig. 4A). Compared to CD15⁻ cells, CD15⁺ cells formed more spheres, and pyrvinium reduced these numbers (Fig. 4B). Furthermore, linking CD15^+^ self-renewal to WNT signaling, similar to pyrvinium, a compound that blocks β-catenin/LEF1 interaction, PKF115-584 [27], impaired sphere formation in similarly sorted cells, while SHH inhibition by vismodegib failed to do so (Fig. 4B). These findings suggest that pyrvinium targets a WNT-driven CD15⁺ population. Gene expression analysis further supported this idea, as CD15-sorted cells showed elevated expression of multiple WNT target genes compared to CD15⁻ cells, while SHH target gene levels were similar (Fig. 4C). Furthermore, treatment of MPC cultures with pyrvinium reduced the CD15⁺ cell population (Fig. 4D). Like pyrvinium, PKF115-584 reduced CD15^+^ cell numbers, whereas vismodegib had no effect (Fig. 4E). Together, these results suggest that pyrvinium targets a WNT-driven CD15⁺ cell population critical for self-renewal.

A Sox2⁺/CD15⁺ population was previously mentioned in the literature [28], but its regulation remains unclear. Given its ability to deplete both Sox2⁺ and CD15⁺ cells, we asked whether the CD15⁺ cells targeted by pyrvinium also express Sox2. Flow cytometry analyses showed that most CD15⁺ cells in MPC cultures are Sox2⁺, and pyrvinium reduced the number of these double-positive cells (Fig. 4F). WNT inhibition with PKF115-584 similarly reduced Sox2⁺ cells (Fig. 4G) and Sox2⁺/CD15⁺ cells (Fig. 4H), whereas vismodegib did not (Fig. 4G, H). In contrast, Gli inhibition by targeting BET reduced Sox2⁺ cells, but did not affect CD15⁺ or Sox2⁺/CD15⁺ cells (Fig. 4I). Together, these findings suggest that pyrvinium depletes Smo inhibitor-resistant, Gli1-driven Sox2⁺ cells, including a WNT-driven CD15⁺ subset (Fig. 4J).

### CK1α agonists reduce MB stemness

**Fig. 5:**
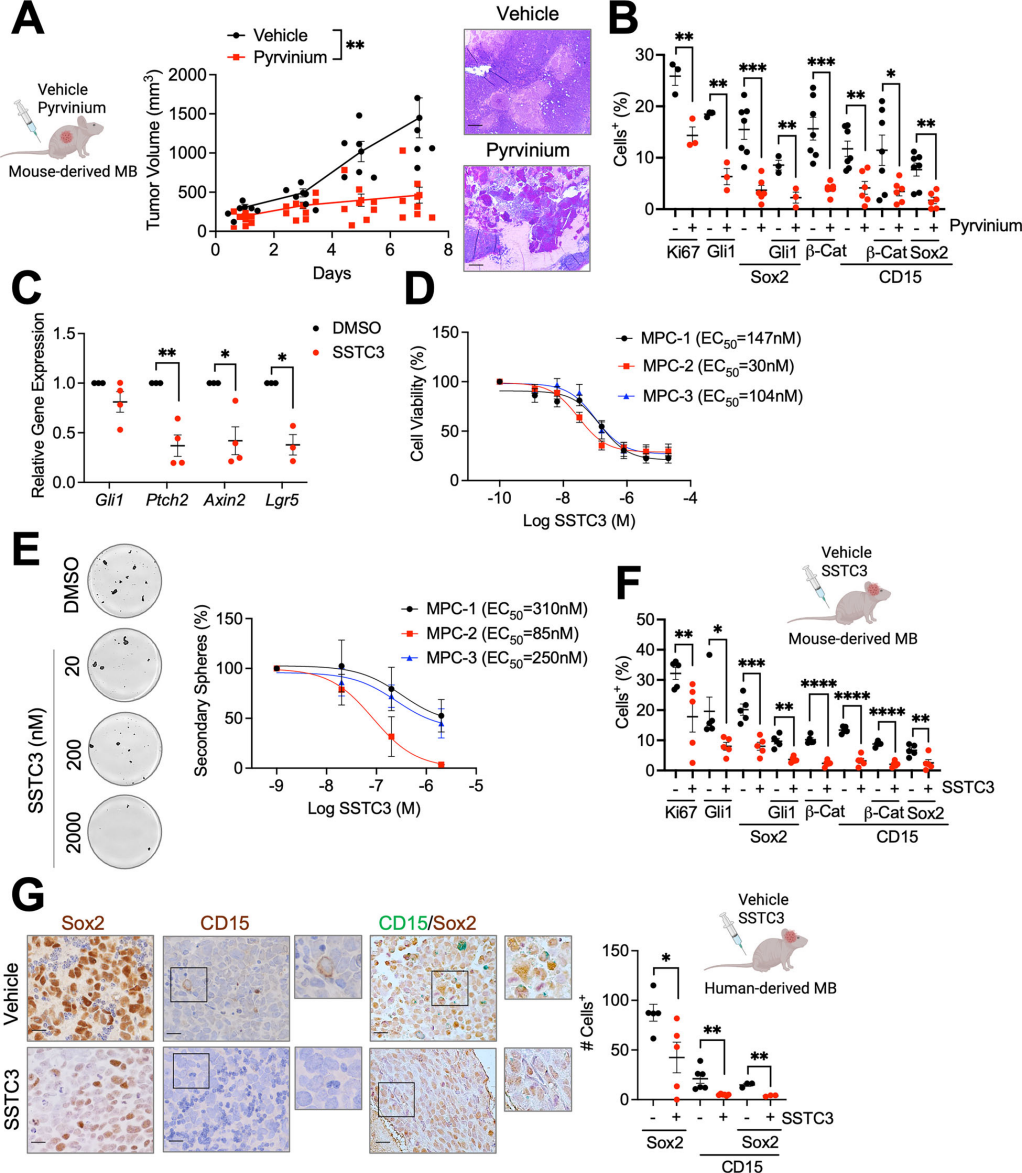
CK1α agonists reduce MB stemness. **A** *Ptch1-LacZ, Trp53-KO* MB cells were subcutaneously implanted. Once tumors reached ~200 mm³, mice were treated with pyrvinium (0.8 mg/kg, s.c., q.o.d.) or vehicle, and tumor volumes were measured over time. Representative H&E staining (scale bar 500 µm) at day 7 is shown. **B** Mice harboring similar tumors received three doses of pyrvinium, and cells positive for the indicated markers were quantified by flow analysis. **C** MPC-2 cultures treated with 1000 nM SSTC3 for 24 h were analyzed by RT-qPCR for SHH and WNT target gene expression. **D** Similar cultures were exposed to increasing SSTC3 concentrations, and cell viability was measured by MTT assay 72 h later. **E** Similar cultures were allowed to form secondary spheres after 24 h SSTC3 treatment. **F** Mice were orthotopically implanted with *Ptch1-LacZ; Trp53-KO* MB cells, then treated with SSTC3 (10 mg/kg, i.p., q.d.) for 3 days starting 10 days post-surgery. Brains were harvested, and numbers of cells positive for the indicated markers were quantified by flow cytometry. **G** Mice with orthotopic SJSHHMB-14-7196 tumors (grown for 30 days) were treated with SSTC3 (10 mg/kg, i.p., q.d.) for 3 days before IHC staining for Sox2 and CD15. Representative images (scale bar 20 µm) are shown. Unless noted, mean ± SEM of data normalized to DMSO were analyzed using an unpaired *t* test. All EC_50_s were calculated using non-linear regression analyses. *P < 0.05, **P < 0.01, ***P < 0.001, ****P < 0.0001.

Given pyrvinium’s ability to deplete Sox2⁺ and CD15⁺ cells in MPC cultures, we tested whether it does the same in vivo. Mice were implanted subcutaneously with *Ptch1-LacZ, Trp53-KO* MB cells, and allowed to form tumors. Because of its poor systemic bioavailability [29], pyrvinium was administered subcutaneously near the tumor site [13]. This local dosing suppressed tumor growth (Fig. 5A and Supplemental Fig. 2A), reduced proliferative index, and decreased the number of Gli1⁺ and Sox2⁺ cells, including double-positive ones (Fig. 5B and Supplemental Fig. 2B). Pyrvinium also depleted CD15⁺ and β-catenin⁺ cells, including a WNT-labeled CD15⁺ subset, and diminished the Sox2⁺/CD15⁺ population within these tumors (Fig. 5B and Supplemental Fig. 2B).

Pyrvinium’s poor bioavailability limits its use to subcutaneous tumor models [29]. To test whether CK1α agonists reduce MB stemness in orthotopic MB, we used a brain-permeable derivative of pyrvinium, SSTC3 [14, 17]. Like pyrvinium, SSTC3 suppressed SHH and WNT target gene expression (Fig. 5C), and reduced cell viability (Fig. 5D) and self-renewal (Fig. 5E) in MPC cultures. In mice bearing orthotopic *Ptch1-LacZ, Trp53-KO* tumors, SSTC3 reduced the proliferation index and the numbers of cells labeled for SHH as well as for WNT markers (Fig. 5F and Supplemental Fig. 3A). SSTC3 also reduced overall Sox2⁺ cells, including Gli1⁺ ones, and decreased CD15⁺, CD15⁺/β-catenin⁺, as well as Sox2⁺/CD15⁺ populations (Fig. 5F and Supplemental Fig. 3A). To determine whether the efficacy of CK1α agonists goes beyond murine models, we tested SSTC3 in a *TP53*-mutant SHH PDOX model. While we previously showed that SSTC3 debulks *TP53*‑mutant SHH tumors and prolongs survival in mice harboring these xenografts [14], we had not evaluated its effects on stemness markers. Here, we show that SSTC3 reduces Sox2⁺, CD15⁺, and Sox2⁺/CD15⁺ populations even in PDOX tumors (Fig. 5G and Supplemental Fig. 3B), despite a higher baseline of Sox2⁺ cells compared to mouse-derived models, supporting efficacy across systems.

### Pyrvinium reduces MB relapse risk

**Fig. 6:**
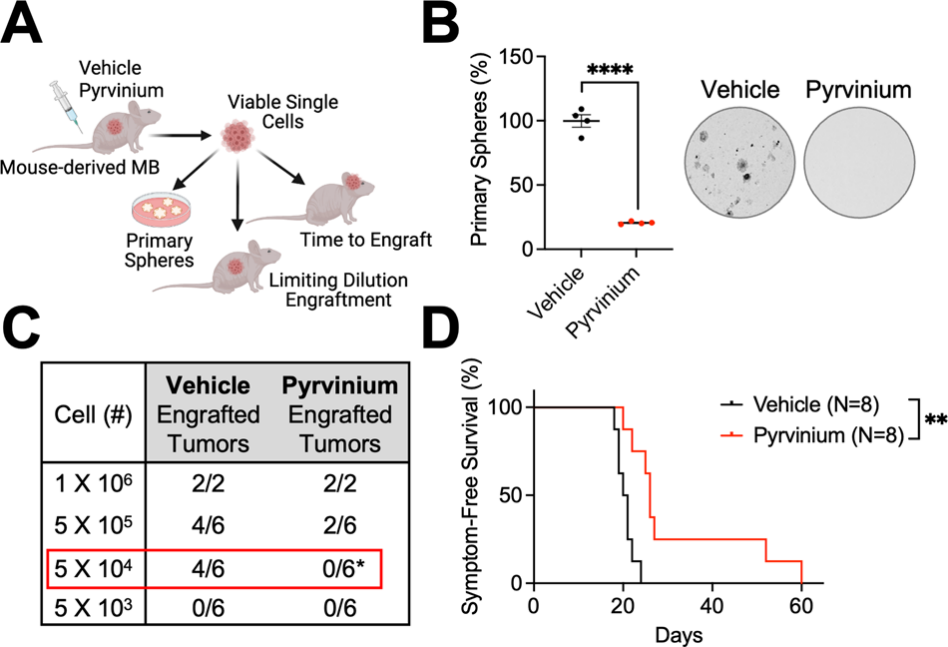
Pyrvinium reduces MB relapse risk. **A** Schematic of relapse-risk protocols: mice bearing subcutaneous *Ptch1-LacZ, Trp53-KO* MB received 3 doses of pyrvinium before preparing single-cell suspensions for primary sphere assays or subcutaneous and orthotopic engraftment. **B** Quantification and representative images of primary spheres from vehicle- and pyrvinium-treated tumors are shown. The data were normalized to one vehicle-treated animal and analyzed using an unpaired *t* test. **C** Tumor engraftment frequency of limiting cell numbers was analyzed with a one-sided *χ*^2^ test. **D** Time to relapse was assessed by symptom-free survival and analyzed by Log-rank (Mantel–Cox) tests. Unless noted, mean ± SEM of data. *P < 0.05, **P < 0.01, ***P < 0.001, ****P < 0.0001.

Based on their ability to reduce MB stemness and ex vivo self-renewal, we next examined whether CK1α agonists would also impair tumor-propagating potential using relapse-risk protocols in which primary sphere formation is assessed along with secondary tumor engraftment and time to engraftment (Fig. 6A). Due to its FDA approval and ongoing cancer clinical trials (NCT05055323, NCT06782048, NCT06590454), we focused these studies on pyrvinium. In primary sphere formation assays, in which equal numbers of viable cells were plated and allowed to form spheres, results showed that pyrvinium-treated tumors fail to propagate in culture (Fig. 6B). Similarly, limiting dilution assays in which equal numbers of viable tumor cells were subcutaneously re-implanted showed differences in engraftment ability between vehicle and pyrvinium-treated tumors (Fig. 6C). Moreover, orthotopic re-engraftment showed that tumors derived from pyrvinium-treated residual disease took longer to establish (Fig. 6D). These results show pyrvinium’s potential to reduce SHHα MB relapse risk.

## Discussion

Here, we demonstrate that compounds activating CK1α attenuate MB stemness and, consequently, reduce relapse risk in animal models. Our findings suggest that these drugs function as dual inhibitors, simultaneously blocking Gli and WNT signaling, key drivers of Sox2⁺ cell proliferation and CD15⁺ self-renewal, respectively. The ability of CK1α agonists to attenuate MB stemness is particularly critical in SHHα MB, which accounts for ~75% of SHH MB recurrences [3], and shows 0–41% 5-year-survival after relapse [3, 5]. While we cannot exclude the possibility that pyrvinium and SSTC3 also attenuate stemness in *TP53*-wild-type SHH MB, this study was focused on the mutant subtype due to the clinical need they represent.

We previously showed that Sox2⁺ MB cells depend on the activation of Gli downstream of Smo, and that these cells are susceptible to depletion by BET inhibitors, which block Gli transcriptional activity [30, 31]. However, efforts to translate these findings have been limited by the clinical toxicity of BET inhibitors [32] and the recent termination of a pediatric brain tumor trial testing them (NCT03936465). Dose-limiting toxicities of BET inhibitors underscores the need for alternative approaches to suppress Gli transcriptional activity. Because Gli proteins are transcription factors and therefore difficult to target directly [33], we focused instead on regulatory mechanisms controlling Gli stability. Since CK1α phosphorylates Gli, priming it for degradation [11], we tested pyrvinium, a previously characterized CK1α agonist [12], in MB cultures enriched for Sox2⁺ cells [9]. Pyrvinium suppressed SHH signaling and reduced the proliferation of Sox2⁺ cells. Thus, this drug shows efficacy like that of BET inhibitors, but with the potential advantage of lower toxicity.

Besides breast cancer [34], CK1α agonists have been reported to attenuate glioblastoma stemness by targeting CD133⁺ cells [35]. Although early work described CD133⁺ MB cells as the self-renewing pool [36], subsequent studies pointed to CD15⁺ cells instead [24–26]. Thus, based on our data showing that pyrvinium blocks MB self-renewal, we studied whether pyrvinium targets CD15⁺ cells. Here, we showed that pyrvinium depletes a self-renewing CD15^+^ cell pool, and it does so by targeting WNT signaling. This aligns with previous evidence implicating WNT signaling in regulating cancer stemness [37]. However, while previous works implicated CD15 cells with WNT signaling in retina progenitors [38], to our knowledge, this is the first study to link CD15⁺ cells with WNT signaling in cancer. Mechanistically, we found that, similar to previous observations [22], WNT activation in SHHα MPC cultures is associated with the loss of p53*/miR-34a*-mediated repression of WNT components such as LEF1. While additional studies are needed to clarify why WNT signaling is specifically required in CD15⁺ cells or which other WNT signaling components are repressed by *miR-34a* in MB, our findings identified a connection between p53 loss, WNT signaling, and CD15-driven MB self-renewal.

Our data support that pyrvinium exerts its effects on stemness by inhibiting Gli and WNT signaling. However, pyrvinium has been reported to inhibit AKT signaling [39], a pathway known to promote survival and maintenance of tumor stem-like cells [40], including those in MB [41]. Similarly, pyrvinium can attenuate STAT3 activity [42, 43], which is a critical regulator of stemness and self-renewal in multiple cancers [44], including MB [45]. Thus, the anti-stemness effects of pyrvinium may involve the suppression of a broader group of oncogenic signaling programs that converge on regulating stemness. Additionally, pyrvinium has been shown to preferentially accumulate in the mitochondria of tumor cells [46], where it disrupts mitochondrial respiration [29, 47] and impairs mitochondrial gene expression [48]. Notably, tumor stem-like cells exhibit heightened mitochondrial requirements [49], providing an additional basis for the preferential targeting of these populations by pyrvinium.

While our work primarily focused on pyrvinium due to its FDA approval and its use in recently launched trials for pancreatic and gastric cancer (NCT05055323, NCT06782048, NCT06590454), it is important to note that pyrvinium does not cross the blood-brain barrier. Although our ex vivo pyrvinium concentrations are comparable to those used in other tumor models [13, 16, 34, 35, 39, 42, 43, 46, 48, 50], including those supporting its translation [48, 51], its lack of brain permeability prevents direct in vivo dose comparisons in MB and limits its direct translational potential for these tumors. While strategies such as intrathecal drug administration could be considered, a more promising approach is the development of derivatives with improved brain permeability. SSTC3, evaluated here in two different orthotopic SHHα MB models, serves as a useful brain‑penetrant tool compound. SSTC3 reduced stemness markers, and as shown in our previous work also prolonged survival in SHH MB orthotopic models, including in a *TP53*-mutant PDOX [14]. Although we initially attributed the survival advantage of SSTC3-treated mice to tumor debulking, our new data suggest that it may in addition result from its effects on tumor stemness. Together, these findings suggest that the efficacy of CK1α agonists extends to stemness regulation and supports their further development, particularly brain-permeable derivatives, for very high-risk SHHα MB patients.

## Supplementary information

Supplemental Figures

Supplemental Methods

Uncropped Blots

## Data Availability

All data presented in this manuscript will be made available upon request. Uncropped versions of all immunoblotting images in this manuscript can be found in Supplemental Material.

## References

[1] Louis DN, Perry A, Reifenberger G, von Deimling A, Figarella-Branger D, Cavenee WK, et al. The 2016 World Health Organization classification of tumors of the central nervous system: a summary. Acta Neuropathol. 2016;131(6):803–20. 27157931 10.1007/s00401-016-1545-1

[2] Northcott PA, Korshunov A, Witt H, Hielscher T, Eberhart CG, Mack S, et al. Medulloblastoma comprises four distinct molecular variants. J Clin Oncol. 2011;29(11):1408–14. 20823417 10.1200/JCO.2009.27.4324 PMC4874239

[3] Tabori U, Baskin B, Shago M, Alon N, Taylor MD, Ray PN, et al. Universal poor survival in children with medulloblastoma harboring somatic TP53 mutations. J Clin Oncol. 2010;28(8):1345–50. 20142599 10.1200/JCO.2009.23.5952

[4] Hill RM, Kuijper S, Lindsey JC, Petrie K, Schwalbe EC, Barker K, et al. Combined MYC and P53 defects emerge at medulloblastoma relapse and define rapidly progressive, therapeutically targetable disease. Cancer Cell. 2015;27(1):72–84. 25533335 10.1016/j.ccell.2014.11.002 PMC4297293

[5] Zhukova N, Ramaswamy V, Remke M, Pfaff E, Shih DJ, Martin DC, et al. Subgroup-specific prognostic implications of TP53 mutation in medulloblastoma. J Clin Oncol. 2013;31(23):2927–35. 23835706 10.1200/JCO.2012.48.5052 PMC4878050

[6] Ramaswamy V, Remke M, Bouffet E, Bailey S, Clifford SC, Doz F, et al. Risk stratification of childhood medulloblastoma in the molecular era: the current consensus. Acta Neuropathol. 2016;131(6):821–31. 27040285 10.1007/s00401-016-1569-6 PMC4867119

[7] Cavalli FMG, Remke M, Rampasek L, Peacock J, Shih DJH, Luu B, et al. Intertumoral heterogeneity within medulloblastoma subgroups. Cancer Cell. 2017;31(6):737–54 e6. 28609654 10.1016/j.ccell.2017.05.005 PMC6163053

[8] Huang GH, Xu QF, Cui YH, Li N, Bian XW, Lv SQ. Medulloblastoma stem cells: promising targets in medulloblastoma therapy. Cancer Sci. 2016;107(5):583–9. 27171351 10.1111/cas.12925 PMC4970825

[9] Swiderska-Syn M, Mir-Pedrol J, Oles A, Schleuger O, Salvador AD, Greiner SM, et al. Noncanonical activation of GLI signaling in SOX2(+) cells drives medulloblastoma relapse. Sci Adv. 2022;8(29):eabj9138. 35857834 10.1126/sciadv.abj9138 PMC9299538

[10] Sun Y, Han J, Wang Z, Li X, Sun Y, Hu Z. Safety and efficacy of bromodomain and extra-terminal inhibitors for the treatment of hematological malignancies and solid tumors: a systematic study of clinical trials. Front Pharm. 2020;11:621093. 10.3389/fphar.2020.621093 PMC7870522 33574760

[11] Robbins DJ, Fei DL, Riobo NA. The Hedgehog signal transduction network. Sci Signal. 2012;5(246):re6. 23074268 10.1126/scisignal.2002906 PMC3705708

[12] Thorne CA, Hanson AJ, Schneider J, Tahinci E, Orton D, Cselenyi CS, et al. Small-molecule inhibition of Wnt signaling through activation of casein kinase 1alpha. Nat Chem Biol. 2010;6(11):829–36. 20890287 10.1038/nchembio.453 PMC3681608

[13] Li B, Fei DL, Flaveny CA, Dahmane N, Baubet V, Wang Z, et al. Pyrvinium attenuates Hedgehog signaling downstream of smoothened. Cancer Res. 2014;74(17):4811–21. 24994715 10.1158/0008-5472.CAN-14-0317 PMC4321822

[14] Rodriguez-Blanco J, Li B, Long J, Shen C, Yang F, Orton D, et al. A CK1alpha activator penetrates the brain and shows efficacy against drug-resistant metastatic medulloblastoma. Clin Cancer Res. 2019;25(4):1379–88. 30487124 10.1158/1078-0432.CCR-18-1319 PMC7142219

[15] Shen C, Nayak A, Melendez RA, Wynn DT, Jackson J, Lee E, et al. Casein kinase 1alpha as a regulator of Wnt-driven cancer. Int J Mol Sci. 2020;21(16):5940. 32824859 10.3390/ijms21165940 PMC7460588

[16] Li B, Flaveny CA, Giambelli C, Fei DL, Han L, Hang BI, et al. Repurposing the FDA-approved pinworm drug pyrvinium as a novel chemotherapeutic agent for intestinal polyposis. PLoS ONE. 2014;9(7):e101969. 25003333 10.1371/journal.pone.0101969 PMC4086981

[17] Li B, Orton D, Neitzel LR, Astudillo L, Shen C, Long J, et al. Differential abundance of CK1alpha provides selectivity for pharmacological CK1alpha activators to target WNT-dependent tumors. Sci Signal. 2017;10(485):eaak9916. 28655862 10.1126/scisignal.aak9916 PMC5555225

[18] Rodriguez-Blanco J, Pednekar L, Penas C, Li B, Martin V, Long J, et al. Inhibition of WNT signaling attenuates self-renewal of SHH-subgroup medulloblastoma. Oncogene. 2017;36(45):6306–14. 28714964 10.1038/onc.2017.232 PMC5680121

[19] Peterson K, Turos-Cabal M, Salvador AD, Palomo-Caturla I, Howell AJ, Vieira ME, et al. Mechanistic insights into medulloblastoma relapse. Pharm Ther. 2024;260:108673. 10.1016/j.pharmthera.2024.108673 PMC11270902 38857789

[20] Lee CH, Yu CC, Wang BY, Chang WW. Tumorsphere as an effective in vitro platform for screening anti-cancer stem cell drugs. Oncotarget. 2016;7(2):1215–26. 26527320 10.18632/oncotarget.6261 PMC4811455

[21] Fabian MR, Sonenberg N, Filipowicz W. Regulation of mRNA translation and stability by microRNAs. Annu Rev Biochem. 2010;79:351–79. 20533884 10.1146/annurev-biochem-060308-103103

[22] Cha YH, Kim NH, Park C, Lee I, Kim HS, Yook JI. MiRNA-34 intrinsically links p53 tumor suppressor and Wnt signaling. Cell Cycle. 2012;11(7):1273–81. 22421157 10.4161/cc.19618

[23] Cadigan KM, Waterman ML. TCF/LEFs and Wnt signaling in the nucleus. Cold Spring Harb Perspect Biol. 2012;4(11):a007906. 23024173 10.1101/cshperspect.a007906 PMC3536346

[24] Fox N, Damjanov I, Knowles BB, Solter D. Immunohistochemical localization of the mouse stage-specific embryonic antigen 1 in human tissues and tumors. Cancer Res. 1983;43(2):669–78. 6129058

[25] Read TA, Fogarty MP, Markant SL, McLendon RE, Wei Z, Ellison DW, et al. Identification of CD15 as a marker for tumor-propagating cells in a mouse model of medulloblastoma. Cancer Cell. 2009;15(2):135–47. 19185848 10.1016/j.ccr.2008.12.016 PMC2664097

[26] Ward RJ, Lee L, Graham K, Satkunendran T, Yoshikawa K, Ling E, et al. Multipotent CD15+ cancer stem cells in patched-1-deficient mouse medulloblastoma. Cancer Res. 2009;69(11):4682–90. 19487286 10.1158/0008-5472.CAN-09-0342

[27] Gandhirajan RK, Staib PA, Minke K, Gehrke I, Plickert G, Schlosser A, et al. Small molecule inhibitors of Wnt/beta-catenin/lef-1 signaling induces apoptosis in chronic lymphocytic leukemia cells in vitro and in vivo. Neoplasia. 2010;12(4):326–35. 20360943 10.1593/neo.91972 PMC2847740

[28] Vanner RJ, Remke M, Gallo M, Selvadurai HJ, Coutinho F, Lee L, et al. Quiescent sox2(+) cells drive hierarchical growth and relapse in sonic hedgehog subgroup medulloblastoma. Cancer Cell. 2014;26(1):33–47. 24954133 10.1016/j.ccr.2014.05.005 PMC4441014

[29] Schultz CW, Nevler A. Pyrvinium pamoate: past, present, and future as an anti-cancer drug. Biomedicines. 2022;10(12):3249. 36552005 10.3390/biomedicines10123249 PMC9775650

[30] Tang Y, Gholamin S, Schubert S, Willardson MI, Lee A, Bandopadhayay P, et al. Epigenetic targeting of Hedgehog pathway transcriptional output through BET bromodomain inhibition. Nat Med. 2014;20(7):732–40. 24973920 10.1038/nm.3613 PMC4108909

[31] Long J, Li B, Rodriguez-Blanco J, Pastori C, Volmar CH, Wahlestedt C, et al. The BET bromodomain inhibitor I-BET151 acts downstream of smoothened protein to abrogate the growth of hedgehog protein-driven cancers. J Biol Chem. 2014;289(51):35494–502. 25355313 10.1074/jbc.M114.595348 PMC4271234

[32] Mita MM, Mita AC. Bromodomain inhibitors a decade later: a promise unfulfilled?. Br J Cancer. 2020;123(12):1713–4. 32989227 10.1038/s41416-020-01079-x PMC7722711

[33] Henley MJ, Koehler AN. Advances in targeting ‘undruggable’ transcription factors with small molecules. Nat Rev Drug Discov. 2021;20(9):669–88. 34006959 10.1038/s41573-021-00199-0

[34] Xu L, Zhang L, Hu C, Liang S, Fei X, Yan N, et al. WNT pathway inhibitor pyrvinium pamoate inhibits the self-renewal and metastasis of breast cancer stem cells. Int J Oncol. 2016;48(3):1175–86. 26781188 10.3892/ijo.2016.3337

[35] Venugopal C, Hallett R, Vora P, Manoranjan B, Mahendram S, Qazi MA, et al. Pyrvinium targets CD133 in human glioblastoma brain tumor-initiating cells. Clin Cancer Res. 2015;21(23):5324–37. 26152745 10.1158/1078-0432.CCR-14-3147

[36] Singh SK, Hawkins C, Clarke ID, Squire JA, Bayani J, Hide T, et al. Identification of human brain tumour initiating cells. Nature. 2004;432(7015):396–401. 15549107 10.1038/nature03128

[37] Katoh M, Katoh M. WNT signaling and cancer stemness. Essays Biochem. 2022;66(4):319–31. 35837811 10.1042/EBC20220016 PMC9484141

[38] Koso H, Ouchi Y, Tabata Y, Aoki Y, Satoh S, Arai K, et al. SSEA-1 marks regionally restricted immature subpopulations of embryonic retinal progenitor cells that are regulated by the Wnt signaling pathway. Dev Biol. 2006;292(1):265–76. 16499901 10.1016/j.ydbio.2005.09.051

[39] Zheng W, Hu J, Lv Y, Bai B, Shan L, Chen K, et al. Pyrvinium pamoate inhibits cell proliferation through ROS-mediated AKT-dependent signaling pathway in colorectal cancer. Med Oncol. 2021;38(2):21. 33554313 10.1007/s12032-021-01472-3 PMC7868320

[40] Xia P, Xu XY. PI3K/Akt/mTOR signaling pathway in cancer stem cells: from basic research to clinical application. Am J Cancer Res. 2015;5(5):1602–9. 26175931 PMC4497429

[41] Hambardzumyan D, Becher OJ, Rosenblum MK, Pandolfi PP, Manova-Todorova K, Holland EC. PI3K pathway regulates survival of cancer stem cells residing in the perivascular niche following radiation in medulloblastoma in vivo. Genes Dev. 2008;22(4):436–48. 18281460 10.1101/gad.1627008 PMC2238666

[42] Feng J, Jiang W, Liu Y, Huang W, Hu K, Li K, et al. Blocking STAT3 by pyrvinium pamoate causes metabolic lethality in KRAS-mutant lung cancer. Biochem Pharm. 2020;177:113960. 32298693 10.1016/j.bcp.2020.113960

[43] Harada Y, Ishii I, Hatake K, Kasahara T. Pyrvinium pamoate inhibits proliferation of myeloma/erythroleukemia cells by suppressing mitochondrial respiratory complex I and STAT3. Cancer Lett. 2012;319(1):83–8. 22210382 10.1016/j.canlet.2011.12.034

[44] Hu Y, Dong Z, Liu K. Unraveling the complexity of STAT3 in cancer: molecular understanding and drug discovery. J Exp Clin Cancer Res. 2024;43(1):23. 38245798 10.1186/s13046-024-02949-5 PMC10799433

[45] Garg N, Bakhshinyan D, Venugopal C, Mahendram S, Rosa DA, Vijayakumar T, et al. CD133(+) brain tumor-initiating cells are dependent on STAT3 signaling to drive medulloblastoma recurrence. Oncogene. 2017;36(5):606–17. 27775079 10.1038/onc.2016.235 PMC5541269

[46] Fu YH, Tseng CY, Lu JW, Lu WH, Lan PQ, Chen CY, et al. Deciphering the role of pyrvinium pamoate in the generation of integrated stress response and modulation of mitochondrial function in myeloid leukemia cells through transcriptome analysis. Biomedicines. 2021;9(12):1869. 34944685 10.3390/biomedicines9121869 PMC8698814

[47] Ishii I, Harada Y, Kasahara T. Reprofiling a classical anthelmintic, pyrvinium pamoate, as an anti-cancer drug targeting mitochondrial respiration. Front Oncol. 2012;2:137. 23061049 10.3389/fonc.2012.00137 PMC3462317

[48] Schultz CW, McCarthy GA, Nerwal T, Nevler A, DuHadaway JB, McCoy MD, et al. The FDA-approved anthelmintic pyrvinium pamoate inhibits pancreatic cancer cells in nutrient-depleted conditions by targeting the mitochondria. Mol Cancer Ther. 2021;20(11):2166–76. 34413127 10.1158/1535-7163.MCT-20-0652 PMC8859979

[49] Sancho P, Barneda D, Heeschen C. Hallmarks of cancer stem cell metabolism. Br J Cancer. 2016;114(12):1305–12. 27219018 10.1038/bjc.2016.152 PMC4984474

[50] Yang J, Lim JT, Santiago Raj PV, Corona MG, Chen C, Khawaja H, et al. Integrative analysis reveals therapeutic potential of pyrvinium pamoate in Merkel cell carcinoma. J Clin Invest. 2025;135(7):e177724. 39933141 10.1172/JCI177724 PMC11957690

[51] Ponzini FM, Schultz CW, Leiby BE, Cannaday S, Yeo T, Posey J, et al. Repurposing the FDA-approved anthelmintic pyrvinium pamoate for pancreatic cancer treatment: study protocol for a phase I clinical trial in early-stage pancreatic ductal adenocarcinoma. BMJ Open. 2023;13(10):e073839. 37848297 10.1136/bmjopen-2023-073839 PMC10582846

