# Supplementary material for: CK1α agonists attenuate medulloblastoma stemness and relapse risk
Source: Cell Death Dis. 2026 Apr 24;17(1):545. doi: 10.1038/s41419-026-08762-6 (PMC13243512; doi:10.1038/s41419-026-08762-6)

**Supplemental Table 1:** List of antibodies used in this manuscript.

**Supplemental Figure 1. A**. Larger field of IF images in Figure 1B. **B**. Single colors for IF staining shown in Figure 1H. **C**. The levels of Gli1 following 24h incubation with increasing concentrations of vismodegib were quantified by immunoblotting. **D**. Levels of indicated proteins were quantified in similarly treated MPC cultures. **E**. Numbers of SOX2-labeled cells were determined by flow cytometry in similarly treated cultures. **F**. The levels of β-catenin following 24h incubation with increasing concentrations of vismodegib were quantified by immunoblotting.


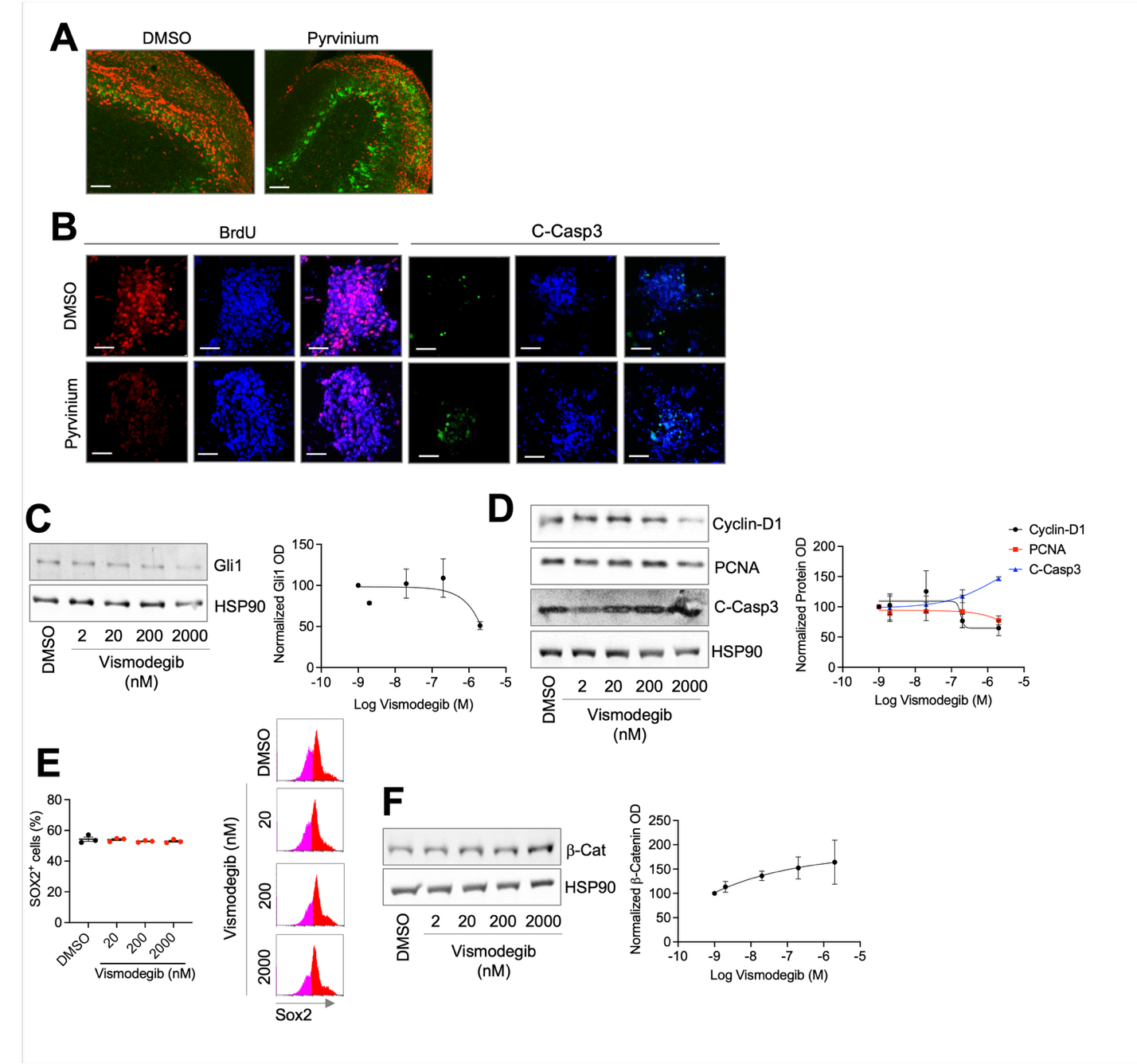


**Supplemental Figure 2. A.** Uncropped images of H&E in Figure 5A. **B**. Representative flow panels for Figure 5B.


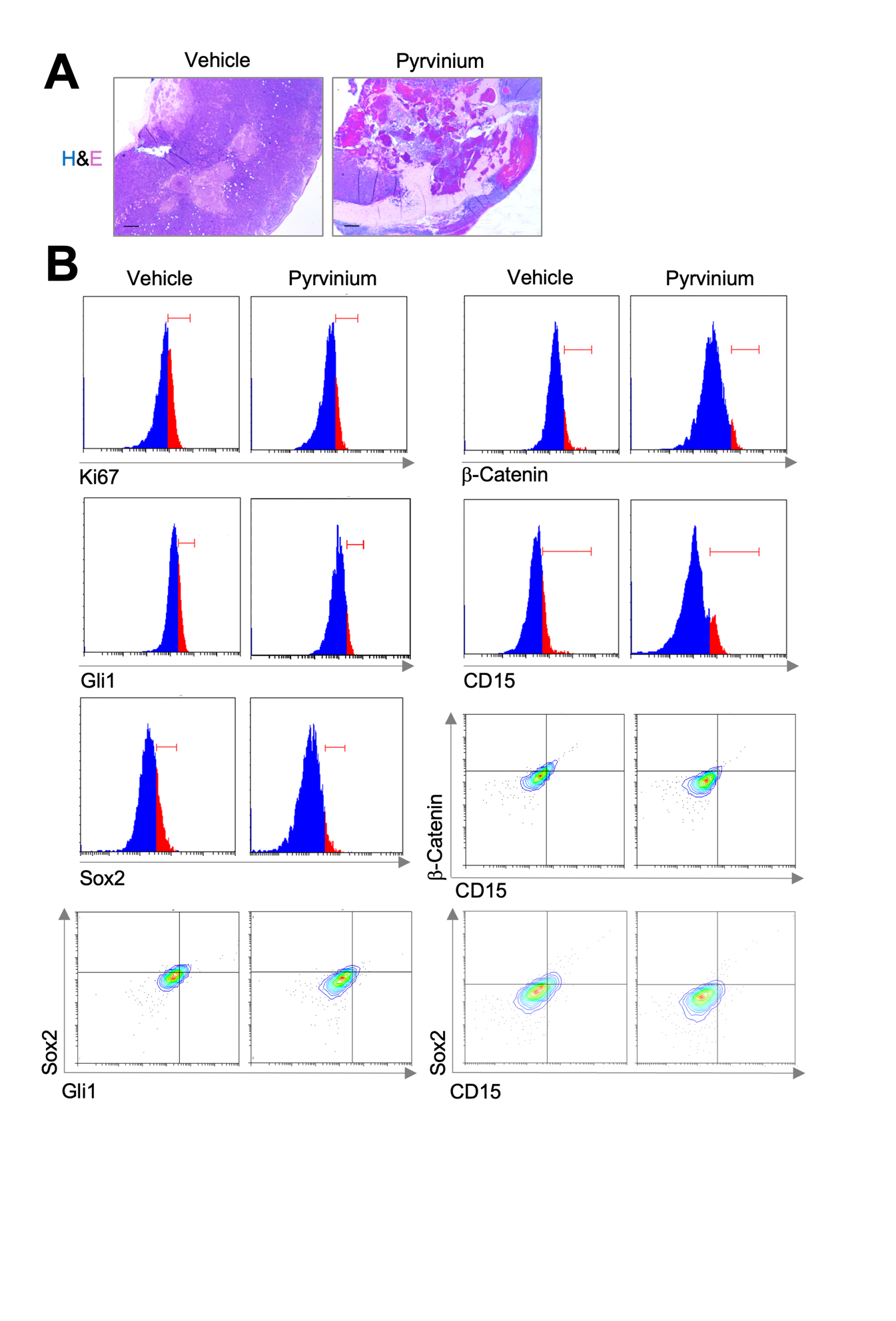


**Supplemental Figure 3. A**. Representative flow panels for Figure 5F. **B**. Larger fields of IHC staining in Figure 5G**.**


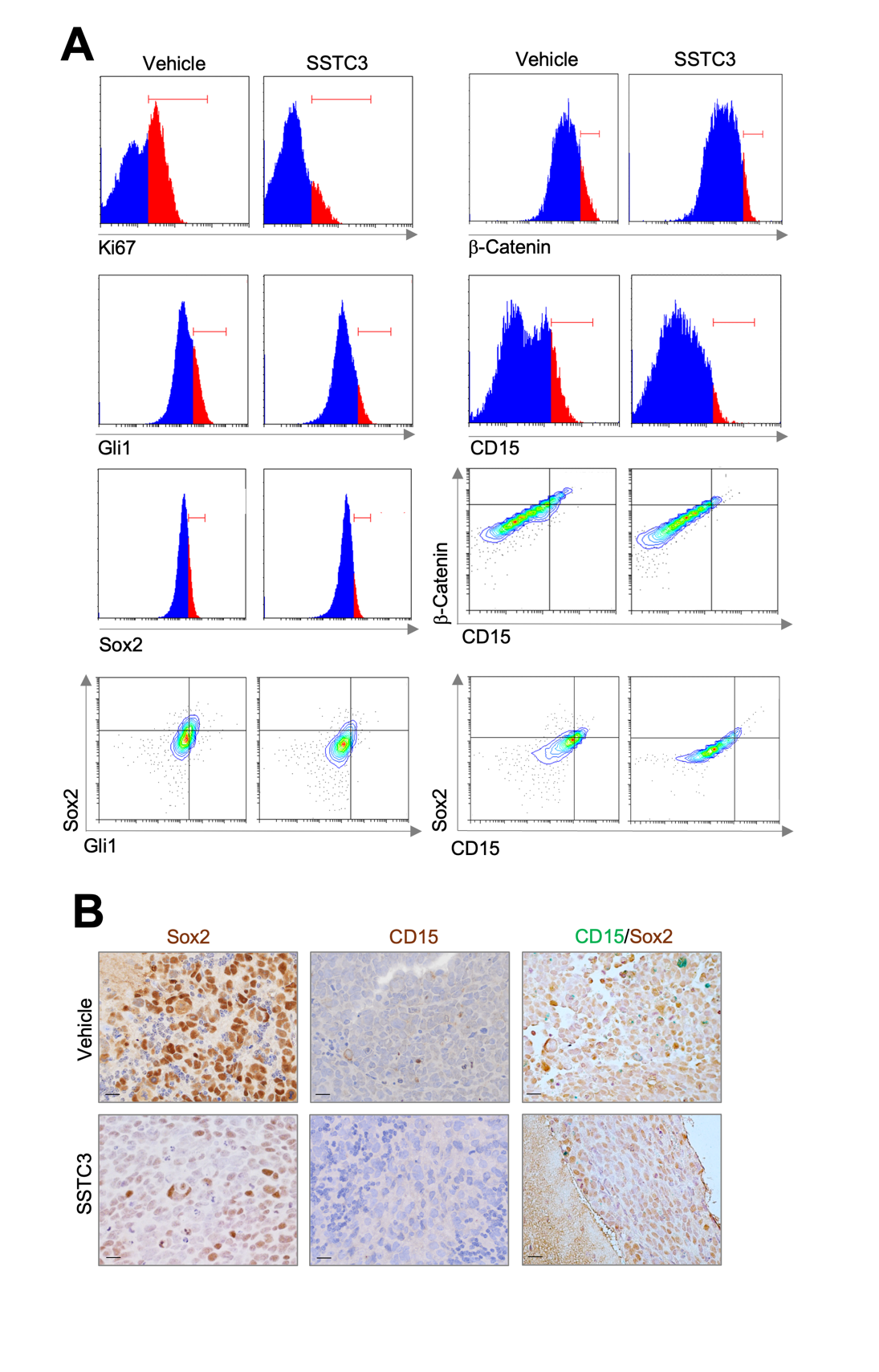

Supplement: Supplementary file 1 — Supplemental Figures [file 41419_2026_8762_MOESM1_ESM.docx]
