# Supplementary material for: CK1α agonists attenuate medulloblastoma stemness and relapse risk
Source: Cell Death Dis. 2026 Apr 24;17(1):545. doi: 10.1038/s41419-026-08762-6 (PMC13243512; doi:10.1038/s41419-026-08762-6)
Supplement: Supplementary file 2 — Supplemental Methods [file 41419_2026_8762_MOESM2_ESM.docx]

**SUPPLEMENTAL METHODS**

**Cell Culture**

MPC-1, MPC-2, and MPC-3 cell suspensions from *Ptch1-LacZ,* *Trp53-KO* tumors, as well as the *Trp53* wild-type MPC-47, were maintained *ex vivo* in Neurobasal serum-free media containing GlutaMAX, B27, N2, EGF (25 ng/ml), FGF (25 ng/ml), and Pen-Strep (all from Invitrogen). *Gli1*-reporter Light2 cells (a gift of Dr. Robbins at Georgetown University) were maintained in DMEM with 10% newborn calf serum (NCS) (R&D Systems), G418 (200 µg/mL), and Zeocin (100 µg/mL). SHH signaling was induced by treating these cells with Smo agonist (SAG) (Sigma-Aldrich) (100 nM) in 0.1% FBS for 24h. *Gli1*-driven firefly luciferase activity was normalized to the Renilla luciferase control, and its activity was determined using a dual luciferase kit (Promega). WNT reporter activity was studied using a cell line containing a T-cell factor (TCF)/LEF luciferase reporter gene, HEK293-STF (ATCC), maintained in DMEM/F12, G-418 (200 µg/mL), and 20% fetal bovine serum (FBS). WNT signaling was induced with 10 mM LiCl (Sigma-Aldrich) and 100 ng/mL WNT3a (R&D Systems), and luciferase data were normalized to protein concentration. Organotypic cultures were obtained from the brains of 8-10-day-old *C57BL/6* mice using a VT1000S vibratome (Leica). Slides were cultured in Basal Medium Eagle (BME) supplemented with glucose (4.5 mg/ml), glutamine, Insulin-Transferrin-Selenium (Gibco), and Pen-Strep in Millicell Cell Culture Inserts (Millipore). Murine cell lines were not subjected to Short Tandem Repeat (STR) profiling. The human HEK293 WNT reporter line was used at low passage shortly after receipt from vendor; no additional STR profiling was performed in-house. All cell lines were routinely tested for mycoplasma contamination.

**Immunohistochemistry**

After in vivo treatment, tumors were harvested, fixed for 48h in 10% neutral buffered formalin (VWR), and embedded in paraffin. Antigen retrieval was performed by steaming tissue sections on glass slides in citrate buffer for 30 minutes before staining using the antibodies and dilutions specified in **Supplemental Table 1**. Horseradish peroxidase (HRP) SignalStain, Boost Detection (Cell Signaling), and Diaminobenzidine (DAB) (DAKO) reagents were applied for antigen detection and counterstained with hematoxylin (Sigma). For double Sox2/CD15 immunohistochemistry (IHC) staining, DAB and a Vina Green chromogen kit (BioCare Medical) were used for antigen detection. IHC images were obtained using an Olympus IX7I or CX43 microscope, and the number of positive cells in tumor tissues was assessed using a blinded visual scoring method.

**Flow and Sorting Analyses**

For extracellular staining, either MPC cultures or cell suspensions from tumor tissues were blocked in 5% bovine serum albumin (BSA) before staining them in 1% BSA with fluorescent-conjugated antibodies (**Supplemental Table 1**). For CD15 staining using unconjugated anti-CD15 antibody, a secondary Alexa-Fluor594 antibody was used for detection. For intracellular staining, once extracellular staining was complete, cells were fixed and permeabilized with Cytofix/Cytoperm solution (BD Biosciences). Cells were then washed in Perm Wash (BD Biosciences), and staining was performed in a similar solution using the indicated antibodies and dilutions (**Supplemental Table 1)**. Flow data acquisition was done on a Beckman Coulter CytoFLEX LX and analyzed by CytoExpert software. A BD fluorescence‑activated cell sorting (FACS) Aria Fusion was utilized for CD15^+^ cell sorting. Cell suspensions from tumors were previously frozen in 10% DMSO to lyse erythroid cells, and Ter-119 was used to ensure their depletion.

**Cell and Molecular Biology**

Cell viability was monitored by the reduction of 3-(4,5-dimethyl-2-thiazolyl) 2,5-diphenyl-2H-tetrazolium bromide (MTT) (Sigma-Aldrich) to formazan. For secondary sphere formation assays, spheres were mechanically disaggregated after treatment, and 1x10^4^ viable cells were allowed to form new spheres in 1 ml of drug-free media for one week. Spheres were stained with MTT for 30 minutes, scanned in a V600 scanner (Epson), and images quantified using ImageJ protocols. For bromodeoxyuridine (BrdU) incorporation and cleaved-Caspase-3 detection by immunofluorescence (IF), MPC cultures were allowed to attach to poly-L-lysine-coated chamber slices (Millipore) for 24h before treatment. Cells were incubated in BrdU (10 μM) (BioLegend) for 2h before fixation in 4% paraformaldehyde (PFA) and staining with antibodies indicated in **Supplemental Table 1**. After treatment, the organotypic slides were similarly incubated with BrdU before being fixed in 4% PFA/4% sucrose and stained with antibodies detecting BrdU and Calbindin (**Supplemental Table 1**). High-magnification images of staining were taken using an Olympus IX7I fluorescent or a Leica TCS SPE confocal microscope, and the number of positive cells per field was assessed using a blinded visual score.

Lipofectamine 2000 (Invitrogen) was used to transfect MPC cultures with *pcDNA3-β-catenin S33Y* plasmid (a gift from Eric Fearon to Addgene, plasmid #19286) using 1 μg DNA in 300,000 cells. Mimic and anti-miR sequences, along with their respective scrambled controls, were purchased from Invitrogen and transfected at 30 nM using similar protocols.

Total RNA was extracted using Trizol (Invitrogen), and the expression of the indicated genes was analyzed by real-time quantitative PCR (RT-qPCR) using TaqMan probes according to the manufacturer’s recommendations (Invitrogen). Data were normalized to the reference gene *Glyceraldehyde-3-phosphate dehydrogenase* (*Gapdh*). Radio-Immunoprecipitation Assay (RIPA) buffer (Thermo Fisher Scientific) supplemented with the HALT protease inhibitor cocktail (Thermo Fisher Scientific) was used for protein extraction, and protein levels were determined by immunoblotting using antibodies and dilutions indicated on **Supplemental Table 1**. Optical density values were quantified in ImageJ and Licor Studio Lite. Heat shock protein 90 (HSP90) and GAPDH were used for loading normalization. mirVana *miRNA* isolation kit (Invitrogen) was utilized for miRNA extraction. Taqman Advanced *miRNA* Assays (Invitrogen) were used to quantify the expression of *miR-34a-5p* and the housekeeping *miRNA miR-361-5p*.
