# Supplementary material for: CK1α agonists attenuate medulloblastoma stemness and relapse risk
Source: Cell Death Dis. 2026 Apr 24;17(1):545. doi: 10.1038/s41419-026-08762-6 (PMC13243512; doi:10.1038/s41419-026-08762-6)

Figure 1C

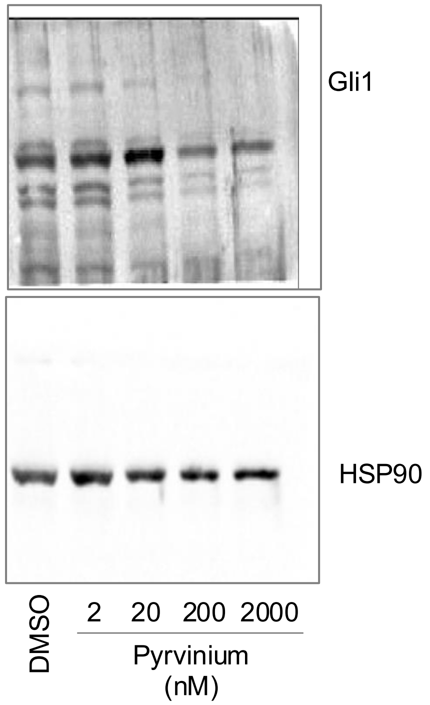

Figure 1D

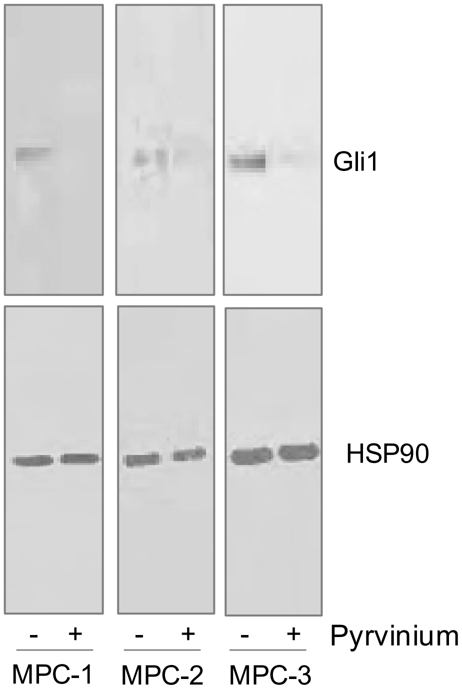

Figure 1I

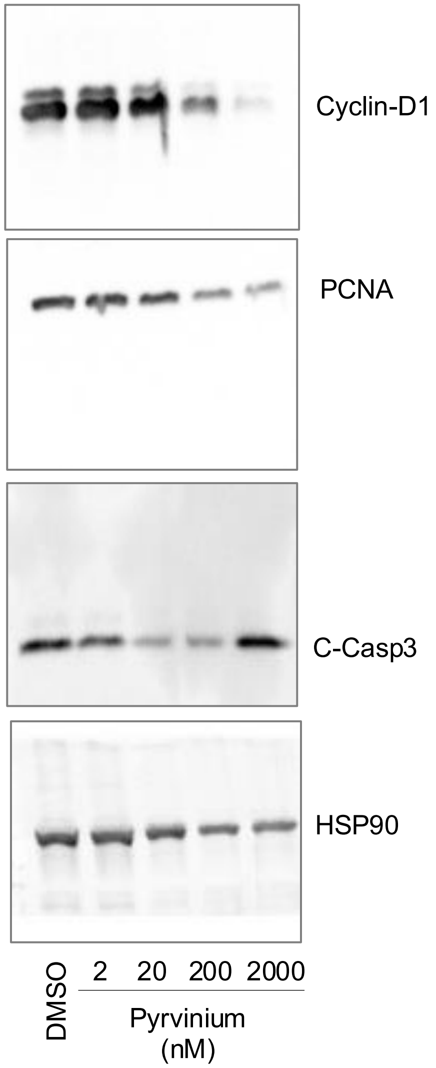

Figure 1J

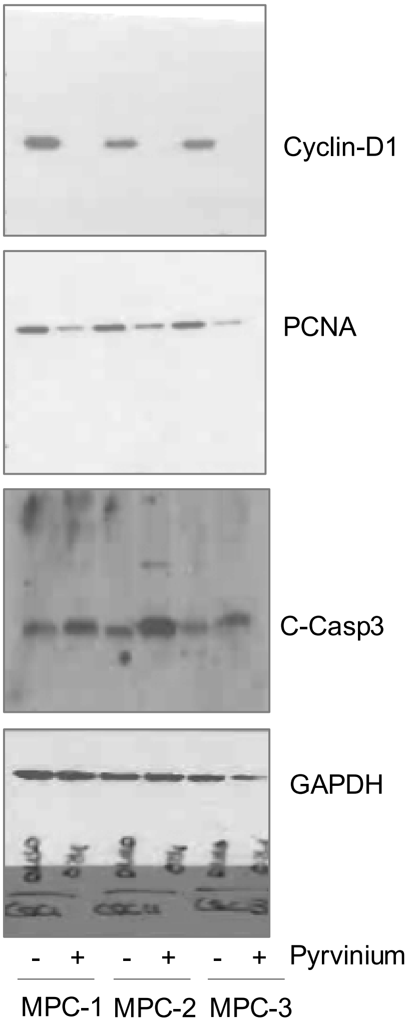

Figure 3B

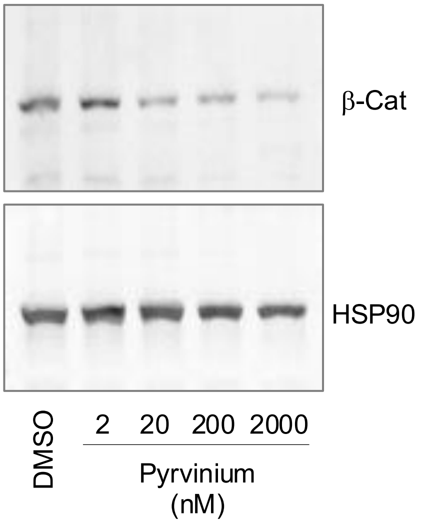

Figure 3C

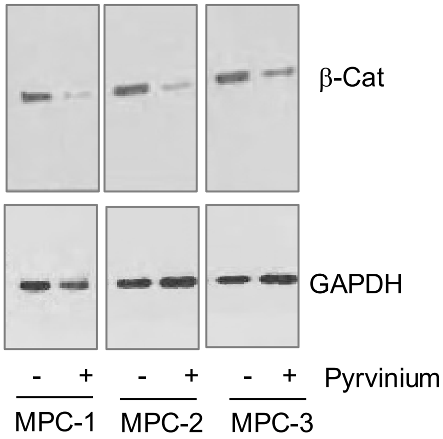

Figure 3H

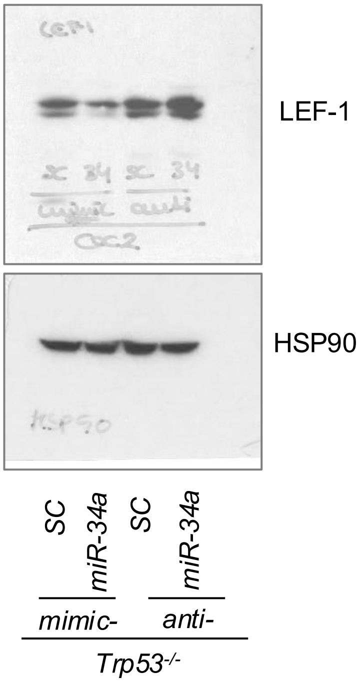

Supplemental Figure 1C

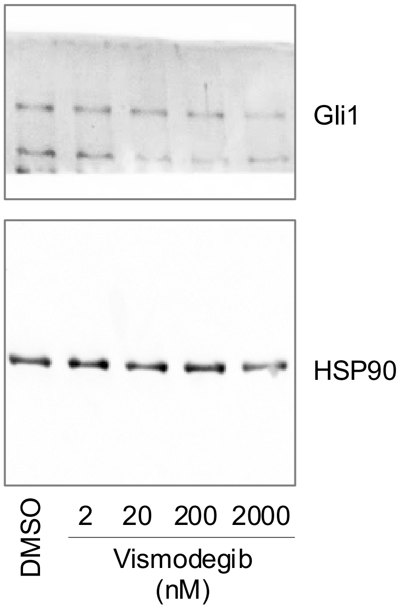

Supplemental Figure 1D

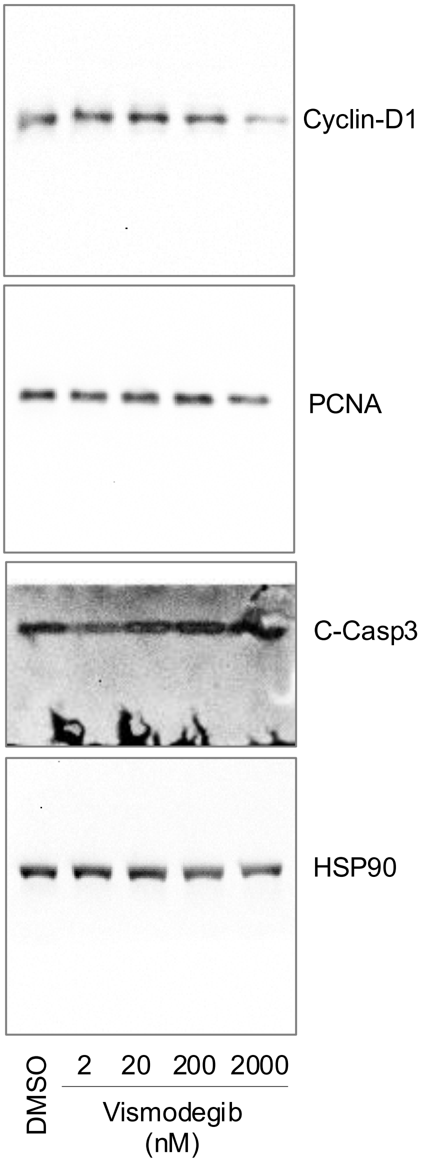

Supplemental Figure 1F

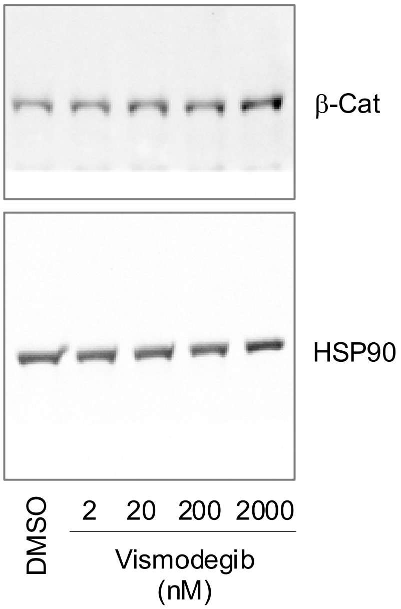

Supplement: Supplementary file 3 — Uncropped Blots [file 41419_2026_8762_MOESM3_ESM.pdf]
